# Apples

**Published:** 1860-10

**Authors:** 


### APPLES.

There is scarcely an article of vegetable food more widely useful and more universally loved than the apple. Why every farmer in the nation has not an apple-orchard where the trees will grow at all, is one of the mysteries. Let every family lay in from two to ten or more barrels, and it will be to them the most economical investment in the whole range of culinaries. A raw mellow apple is digested in an hour and a half; while boiled cabbage requires five hours. The most healthful desert which can be placed on the table, is a baked apple. If taken freely at breakfast with coarse bread and butter, without meat or flesh of any kind, it has an admirable effect on the general system, often removing constipation, correcting acidities, and cooling off febrile conditions, more effectually than the most approved medicines. If families could be induced to substitute the apple, sound, ripe, and luscious, for the pies, cakes, candies, and other sweetmeats with which their children are too often indiscreetly stuffed, there would be a diminution in the sum total of doctors' bills in a single year, sufficient to lay in a stock of this delicious fruit for a whole season's use.

